# Generation of beta-lactoglobulin knock-out goats using CRISPR/Cas9

**DOI:** 10.1371/journal.pone.0186056

**Published:** 2017-10-10

**Authors:** Wenjun Zhou, Yongjie Wan, Rihong Guo, Mingtian Deng, Kaiping Deng, Zhen Wang, Yanli Zhang, Feng Wang

**Affiliations:** Jiangsu Livestock Embryo Engineering Laboratory, Nanjing Agricultural University, Nanjing, Jiangsu, PR, China; Institute of Zoology Chinese Academy of Sciences, CHINA

## Abstract

Goat’s milk, considered a substitute for cow’s milk, has a high nutritional value. However, goat’s milk contains various allergens, predominantly β-lactoglobulin (BLG). In this study, we employed the CRISPR/Cas9 system to target the *BLG* locus in goat fibroblasts for sgRNA optimization and generate *BLG* knock-out goats through co-injection of Cas9 mRNA and small guide RNAs (sgRNAs) into goat embryos at the one-cell stage. We firstly tested sgRNA editing efficiencies in goat fibroblast cells, and approximately 8.00%–9.09% of the cells were modified in single sgRNA-guided targeting experiment. Among the kids, the genome-targeting efficiencies of single sgRNA were 12.5% (10 ng/μL sg1) and 0% (10 ng/μL sg2) and efficiencies of dual sgRNAs were 25.0% (25 ng/μL sg2+sg3 group) and 28.6% (50 ng/μL sg2+sg3 group). Relative expression of *BLG* in *BLG* knock-out goat mammary glands significantly (*p <* 0.01) decreased as well as other milk protein coding genes, such as *CSN1S1*, *CSN1S2*, *CSN2*, *CSN3* and *LALBA* (*p* < 0.05). As expected, BLG protein had been abolished in the milk of the *BLG* knock-out goat. In addition, most of the targeted kids were chimeric (3/4), and their various body tissues were edited simultaneously. Our study thus provides a basis for optimizing the quality of goat milk, which can be applied to biomedical and agricultural research.

## Introduction

The goat (*Capra hirus*) is one of the most important livestock species, providing products such as meat, hides, and milk. Goat’s milk and its byproducts, such as yogurt, cheese, and powder, are important components of the daily human diet in many countries [1]. Furthermore, goats are also used as mammary gland bioreactors in biomedical studies [2].

Goat’s milk has similar nutritional value to cow’s milk, with high percentages of fats and proteins. β-lactoglobulin (BLG) is a major whey protein allergen in goat and other ruminants’ milk [3]. Hydrolysis and heat do not suppress the allergenicity of BLG, and fermentation byproducts increases its immuno-reactivity [4, 5].

Zinc finger nuclease (ZFN) and transcription activator–like effector nucleases (TALENs) are recently developed genomic engineering tools. However, the complexity of protein design and synthesis of ZFN and TALENs had hampered their application in medical and biological research [6, 7]. *Natronobacterium gregoryi* argonaute (NgAgo) is a newly developed DNA guide endonuclease; however, it has been reported that Ago cannot cut the genomic DNA but can knockdown the gene expression[8, 9]. Clustered regularly interspaced short palindromic repeats (CRISPR) are short segments of prokaryotic DNA containing repetitive base sequences; CRISPR functions as an adaptive immune system in prokaryotes and has been adapted for genome editing in eukaryotes [10]. Small guide RNAs (sgRNAs) are used to guide Cas9 protein to specifically cleave DNA strands, causing double-strand breaks that are subsequently repaired through either non-homologous end joining or homology-directed repair mechanisms [11, 12]. Editing of the *BLG* gene was achieved in goat fibroblasts by using Cas9 [13] and TALENs[14], and *BLG* knock-out (KO) cattle has been generated by using ZFN [15]. CRISPR/Cas9 has also been used in knocking out *Myostatin* and *FGF5* in goat [16, 17] and *Myostatin* in sheep [18] via injection of Cas9 mRNA and sgRNA. Thus, to generate *BLG* KO goats for use in our research, we employed the CRISPR/Cas9 system cytoplasmic injection method. We then characterized the changes in the genotype and phenotype during lactation in *BLG* KO goats. These results provided valuable insight into the *BLG* gene in goats and methods of goat milk quality improvement.

## Materials & methods

### Animals

Healthy goats (2 to 3 years old) were selected and housed at the Haimen Goat Research & Development Center in Jiangsu. All protocols involving the use of animals were performed in accordance with the approved Guidelines for Animal Experiments of Nanjing Agricultural University, which were approved by the Animal Care and Use Committee of Nanjing Agricultural University (Approval ID: SYXK2011-0036).

### sgRNA design

**Fig 1:**
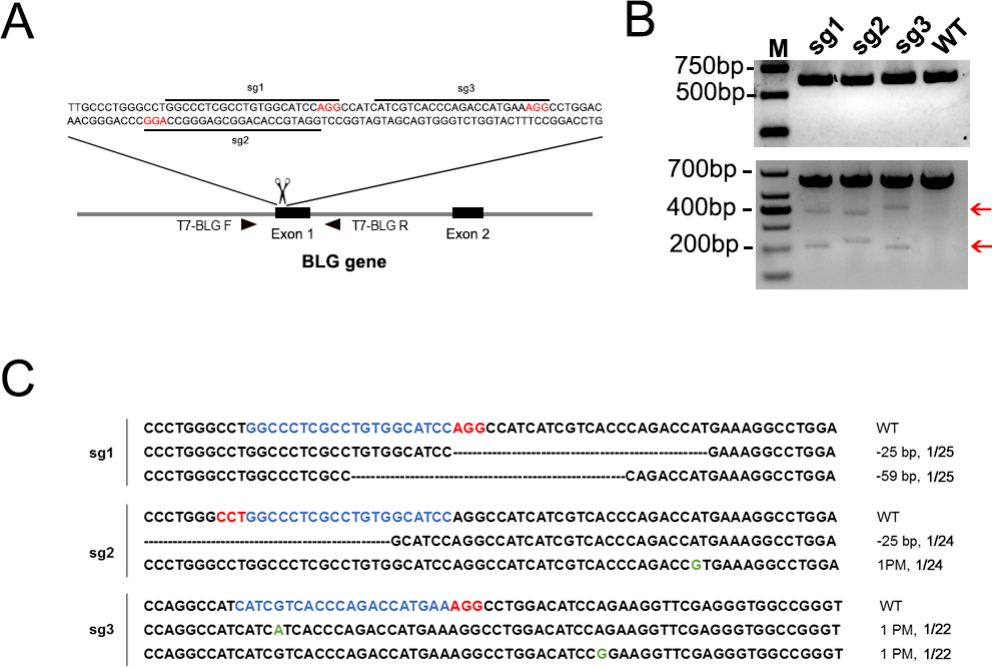
CRISPR/Cas9-mediated modification of the *BLG* locus in fibroblasts. (**A**) Schematic diagram of sgRNA design for the goat *BLG* locus. Primers labeled as “BLG-T7-F” and “BLG-T7-R” were used for the T7E1 cleavage assay at the sg1, sg2, and sg3 target sites. (**B**) Targeting *BLG* loci using single sgRNA by electroporation. Top panel: PCR products of the target region of *BLG* from fibroblasts transfected with a single Cas9-sgRNA plasmid. Bottom panel: T7E1 assay of products shown in the top panel. M, marker; WT, wild-type cells without treatment with Cas9 plasmid. Red arrows indicate the expected cleaved products after T7E1 cleavage assay. (**C**) Sequencing results of sgRNAs targeting *BLG*.

The pX330 plasmid was donated by Libin Cui PhD in the US. The sgRNA was designed using the MIT CRISPR design tool website (http://crispr.mit.edu/). Then sgRNAs were screened by Cas-Offinder and sgRNAs with fewer mismatches were chosen. Three sgRNAs targeting exon 1 of the goat *BLG* gene (GenBank: Z33881) were chosen (Fig 1A). Two of the sgRNAs (sg1, sg3) were on the sense strand, and the third (sg2) was on the antisense strand; a guanine (G) was added at the 5`end of the guide sequence without a guanine at the beginning of the 5`end (S1 Table). The sgRNAs were cloned into pX330 to form the final vectors, Cas9-sg1, Cas9-sg2, and Cas9-sg3.

### Cell culture and transfection

Goat fetal fibroblasts were isolated as described previously [19] and cultured in Dulbecco’s modified Eagle’s medium (Gibco, USA) supplemented with 15% fetal bovine serum (FBS; Gibco) and 1% glutamine (Gibco) in T25 flasks or 6-well plates until reaching 80–90% confluence on the day of transfection. Cells were transfected with either single Cas9-sgRNA plasmid (1 μg/10^5^ cells) or eGFP plasmid (1 μg/10^5^ cells) using a Neon transfection system (Life Technologies, USA) or using Lipofectamine 2000 (Invitrogen, USA), according to the manufacturer’s protocols. Cells were collected for DNA extraction 72 h after transfection.

### *In vitro* transcription

The *in vitro* transcription templates for Cas9 and the sgRNAs were amplified using the T7 promotor–appended primers listed in S3 Table and gel-purified using QiaQuick spin columns (Qiagen, Germany). The Cas9 template was transcribed *in vitro* using a T7 Ultra kit (Ambion, USA), and the sgRNA templates were transcribed *in vitro* using a MEGA shortscript kit (Ambion). The resulting Cas9 mRNA and sgRNAs were then purified using a MEGAclear kit (Ambion).

### Preparation and injection of one-cell embryos

Goats were subjected to a superovulation protocol, as previously described [17]. Briefly, a progesterone sponge was implanted in the vagina for 11 days; when the sponge was removed, the animal was administered 100 IU of prostaglandin (Sansheng, China). The donors received a total of 200 IU of follicle-stimulating hormone (Sansheng) twice daily in a decreasing dose over 3 days (50/50, 25/25, and 15/15 IU), starting 48 h before sponge removal. Then donors were mated at 36 and 48 h after sponge removal. The recipients received 100 IU of pregnant mare serum gonadotropin (Sansheng) 24 h prior to sponge removal. One-cell-stage embryos were flushed from the donor oviducts 72 h after sponge removal. Collected one-cell-stage embryos were then injected with a mixture of Cas9 mRNA and sgRNAs (Table 1). Injected zygotes were then cultured in M2 medium containing 10% FBS (Gibco) at 37°C and 5% CO_2_. Cleaved two-cell to blastocyst stage embryos were transferred into estrous-synchronized recipient goats. Early pregnancy was confirmed by observing the estrous cycling 28–30 days after the embryo transfer.

**Table 1:** Summary of generation of gene-modified goats through micro-injection.

sgRNA(ng/μL)	Cas9 mRNA (ng/μL)	Injected embryos/ Transferred embryos	Recipients/ Pregnancies	Newborns	Targeted (%)
sg1(10)	20	36/27	10/5	8	1 (12.5%)
sg2(10)		48/29	10/5	7	0 (0%)
sg2+sg3 (25+25)	100	59/15	15/3	4	1 (25.0%)
sg2+sg3* (50+50)	200	50/32	32/5	7	2 (28.6%)

*One recipient died after transfer of zygotes. One kid was stillborn, and three died within 1 month after delivery.

### T7 endonuclease 1 (T7E1) cleavage assay and Sanger sequencing of the PCR amplicons

Ear genomic DNA and cell DNA were extracted using a DNA extraction kit (Tiangen, China). The genomic regions surrounding each target site were amplified using PrimerStar HS DNA polymerase (Takara, China) with the primers listed in S3 Table and then purified using a QiaQuick spin column (Qiagen), following the manufacturer’s protocol. A total of 300 ng of the purified PCR product was mixed with NEB buffer 2 and subjected to the re-annealing process to enable heteroduplex formation: 95°C for 10 min, ramping from 95 to 85°C at −2°C/s, 85 to 25°C at −0.25°C/s, and holding at 25°C for 1 min. Next, 0.5 μL of T7E1 (NEB, USA) was added to the products, which were incubated for 30 min and then resolved on a 2.5% agarose gel. The PCR products with mutations detected using the T7E1 cleavage assay were then sub-cloned into the pMD-19T vector (Takara). For each sample, 12 to 25 random colonies were picked for Sanger sequencing.

### Detection of off-target activity

Potential off-targets (OTs) of the sgRNAs were computationally predicted using Cas-Offinder [20], according to the goat genome assembly v2.0 [21]. We selected OTs containing “NGG” or “NAG” protospacer adjacent motifs and fewer than five mismatched nucleotides (S2 Table). The potential OTs of *BLG*-modified goat were screened using the methods described above (S3 Table).

### Gene expression analysis

Total RNA was extracted from the mammary glands of hormonally induced lactating *BLG* KO goat and wild-type (WT) goats two weeks after hormone-induced lactation using an RNA extraction kit (Tiangen). The concentration of extracted RNA was determined using a NanoDrop spectrophotometer (Thermo, 2000C). First-strand cDNAs were generated through reverse transcription using 1 μg of total RNA and oligo-dT primers. The sequences and GenBank accession numbers of the primer sets used to amplify the target genes are listed in S3 Table. qPCR assessment was performed using an Applied Biosystems StepOne^TM^ Real-Time PCR Systems (Applied BioSystems, USA), and fluorescence was detected using SYBR Green (Roche, Germany) in a reaction volume of 20 μL. The quantity of each measured cDNA sample was normalized to the reference gene, GAPDH. Target-gene relative expression was determined using the ΔΔCT method. For ease of comparison, the average expression level of each gene from the control goats was set at 1.00. The primers used were described by Eleni *et al* [22].

### Induction of lactation and isolation of whey protein in goat’s milk

Lactation was hormonally induced in 8-month-old goats according to a previously described protocol [23]. Briefly, the animals were given estradiol (0.25 mg/kg, intramuscularly [IM]; Sansheng) and progesterone (0.75 mg/kg, IM; Sansheng) every other day from the 1^st^ day to the 13^th^ day; prednisolone (0.4 mg/kg, IM; Energy Chemical, China) was given from the 14^th^ day to the 16^th^ day. Daily mammary massage was carried out since the 5^th^ day. Milk components were analyzed using a Julie Z9 milk analyzer (MilkoScope, Bulgaria). The isolation of whey protein in goat’s milk was performed as described previously [14].

### Sodium dodecyl sulfate–polyacrylamide gel electrophoresis (SDS-PAGE), Coomassie blue staining, and western blot

Whey protein in the milk serum was analyzed by SDS-PAGE with Coomassie blue staining. The proteins in the whey were isolated as described above. Samples were denatured in SDS gel-loading buffer at 98°C for 10 min. Total proteins (1 μL milk serum) were separated by 12% SDS-PAGE and stained with Coomassie blue or electrotransferred to polyvinylidene fluoride (PVDF) membranes (Millipore, Billerica, USA). After incubation in blocking buffer (5% BSA in Tris-buffered saline containing 0.1% Tween 20) for 1 h at room temperature, the membrane was incubated overnight at 4°C with a Rabbit anti-BLG primary antibody (Bioss, China, bs-2065R, 1:500 dilution). After washing, the membrane was incubated with a goat anti-rabbit IgG (H + L) secondary antibody (Abcam, USA, ab97051, 1: 5,000 dilution) for 1 h at RT. After washing, the signal was detected using an ECL western blot detection system (Fujifilm, Tokyo, Japan).

### Data analysis

The data were analyzed using one-way analysis of variance, followed by Duncan’s’ multiple comparison test using SPSS software (SPSS Inc., USA). Data were derived from at least three independent experiments. Differences in the relative expression of mammary gland genes were analyzed by Student’s t-test. A value of *p <* 0.05 was considered significant.

## Results

### Efficiency of the CRISPR/Cas9 system in fibroblasts

Three sgRNAs targeting the goat *BLG* locus were designed. To test the genome editing efficiencies of different transfection methods, a 9-kb plasmid containing enhanced green fluorescent protein was transfected into goat fibroblasts using lipofection or electroporation method. Flow cytometry was performed after 48 h, and the result revealed that about 1.4% of the cells were green fluorescence–positive in the lipofection group, and 16.5% of cells were green fluorescence–positive in the electroporation group (S1 Fig). Therefore, we chose the electroporation method for subsequent experiments. To test the editing efficiency of the three sgRNAs, each of the three Cas9-sgRNA plasmids was transfected into goat fibroblasts, and genomic DNA was extracted 72 h after transfection. The target region was PCR amplified and subjected to the T7E1 cleavage assay. Additional bands with expected sizes (sg1: ~396 bp and ~226 bp; sg2: ~382 bp and ~240 bp; sg3: ~425 bp and 197 bp) in the T7E1 cleavage assay indicated that the *BLG* loci were modified in the three populations of transfected cells (Fig 1B).

Sequencing of T-clones showed that the three target sites were edited with indels: 25- and 59-bp deletions at the sg1 target site; a 1-bp point mutation and a 25-bp deletion around the sg2 target site; two 1-bp point mutations near the sg3 target site (Fig 1C). The sequencing results showed short deletions (4 to 59 bp) and point mutations caused by single sgRNA-guided Cas9 editing, however, no insertion was detected in our cell-targeting experiments (Fig 1C). The single sgRNA-targeting efficiencies are 8.00% (2/25), 8.33% (2/24), and 9.09% (2/22) for sg1, sg2, and sg3, respectively.

### Generation of genome-modified goats

To generate *BLG* KO goats, we collected a total of 193 embryos; one-cell embryos were then co-injected with sgRNAs and different concentrations of Cas9 mRNA by cytoplasmic injection. A total of 103 zygotes were transferred into 67 pseudo-pregnant female goats (1.54 embryos per recipient). Among the 67 recipients, 18 pregnancies were established, as determined by estrous cycle observation. After approximately 150 days of gestation, 26 kids were delivered, one (#B1-8 injected with sg1) of which was stillborn and three (#B2-4 injected with 50 ng/μL sg2+sg3, #B2-8 and #B2-10 injected with 100 ng/μL sg2+sg3) of which died within 1 month after birth (Table 1).

**Fig 2:**
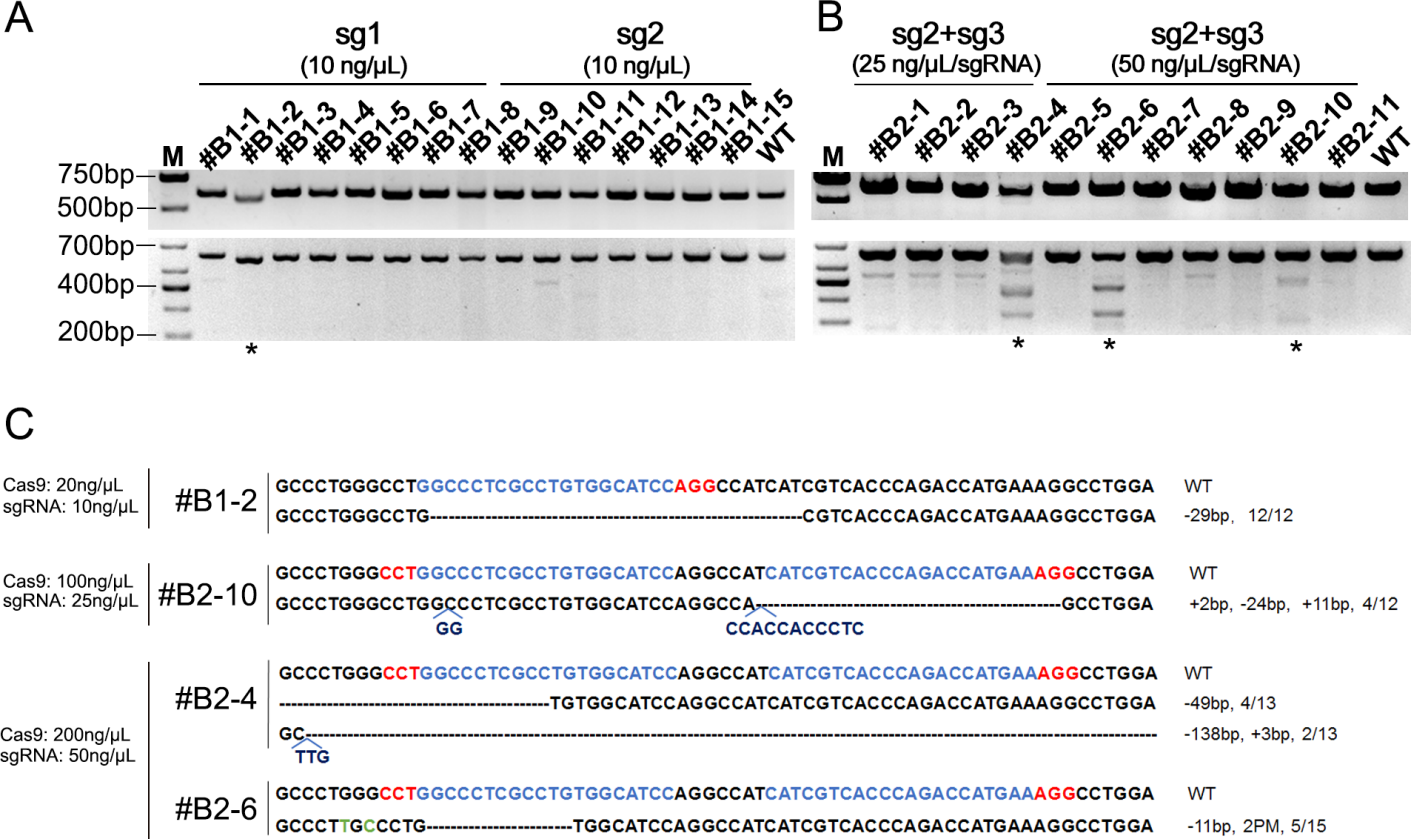
CRISPR/Cas9-mediated one-step generation of *BLG* KO goats. (**A**) Top panel: PCR products of the target region of *BLG* from newborn goats micro-injected with Cas9 and single sgRNA. Bottom panel: Results of T7E1 assay of products shown in the top panel. #B1-1 to #B1-8 were injected with sg1, and #B1-9 to #B1-15 were injected with sg2. (**B**) Top panel: PCR products of the target region of *BLG* from newborn goats micro-injected with Cas9 and dual sgRNAs. Bottom panel: Results of T7E1 assay of products shown in the top panel. #B2-1 to #B2-4 were injected with sg2+sg3 (25 ng/μL/sgRNA), and #B2-5 to #B2-11 were injected with sg2+sg3 (50 ng/μL/sgRNA). (**C**) Sequencing results of *BLG*-modified kids injected with different concentrations. WT, wild-type. PM, point mutation.

Genomic DNA was isolated from the ear of the dead fetus and infant goats for T7E1 assay and Sanger sequencing. The results showed that 15.38% (4/26) of the infants were edited at the *BLG* locus. A lower-molecular-weight band was observed in the PCR product of #B1-2, which indicated a homozygous deletion at the *BLG* locus (Fig 2A). In addition, 12.5% (1/8) of infants in the group co-injected with sg1(10 ng/μL) and Cas9 mRNA (100 ng/μL) exhibited *BLG* modifications at target sites, whereas 0% of infants in the group co-injected with sg2 (10 ng/μL) and Cas9 mRNA (100 ng/μL) were *BLG*-modified. Furthermore, 25.0% of the infants (1/4) were modified by co-injection of sg2 and sg3 (25 ng/μL per sgRNA) with 100 ng/μL Cas9 mRNA, and 28.6% (2/7) were modified in the group co-injected with sg2 and sg3 (50 ng/μL per sgRNA) and 200 ng/μL Cas9 mRNA (Table 1 and Fig 2). Different indel types were observed in our targeted goats, such as more insertions and point mutations in the dual sgRNA-targeted goats and a longer fragment deletion in #B2-4 (Fig 2C). These indels could cause frame shift mutations and changes of protein sequence (S2 Fig).

The bands of unexpected size were observed in the lanes of *#*B1-1, *#*B1-10, *#*B1-11, *#*B2-1 to *#*B2-3, *#*B2-8, and #B2-9 in the T7E1 assay, and resulted from heterozygous genotypes at the *BLG* locus (Z33881 g. 1866 C > T, 2015 G > T, 2391 A > C, S3 Fig).

### Analysis of mammary glands and milk from *BLG*-targeted goats

**Fig 3:**
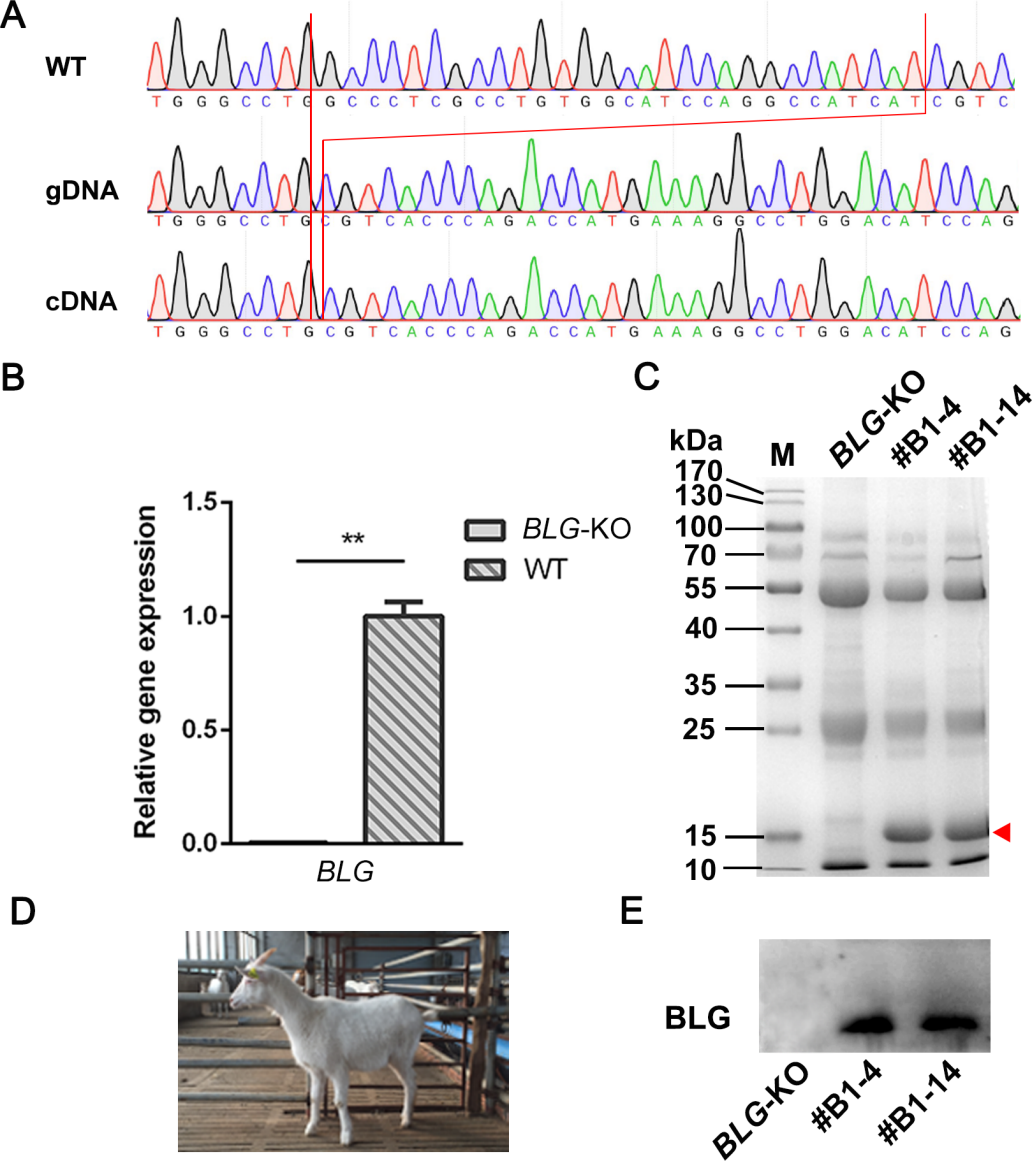
Analysis of genotype and *BLG* expression in milk and mammary gland. (A) Comparison of the *BLG* target region of gDNA in mammary glands of the *BLG* KO (#B1-2) and WT goats, and cDNA of the *BLG* KO goat. Red lines indicate a 29-bp deletion at the *BLG* locus. (B) Analysis of *BLG* expression in mammary glands. Values with two asterisks are significantly different (*p <* 0.01). Three assay replicates were performed for each tissue sample. (C) Analysis of whey protein from hormonally induced goat’s milk. Analysis of whey proteins by Coomassie blue staining after separation by SDS-PAGE. Equal amounts of milk samples were loaded. The red triangle indicates WT BLG bands. (D) A photo of a live targeted goat. (E) Detecting the BLG protein in whey by western blot. *BLG*-KO, the biallelic *BLG* KO goat; #B1-4 and #B1-14, wild-type goats.

To investigate the phenotype change, the *BLG* KO goat #B1-2 and WT goats were hormonally induced for lactation. We then collected the milk and mammary gland tissues. Genomic DNA was extracted from the mammary gland tissues for PCR amplification, and the mRNA was extracted and used for RT-PCR amplification. The genomic *BLG* locus and CDS region of BLG mRNA were amplified and sequenced. The results revealed a 29-bp deletion in #B1-2 *BLG* exon 1, which perfectly matched the previous sequencing results of ear tissue (Fig 3A). Quantitative PCR results showed that *BLG* gene expression in *BLG* KO goats was significantly (*p <* 0.01) lower than in WT goats. (Fig 3C). To investigate the effect of knocking out *BLG* gene on other main milk protein coding genes’ expression level during lactation, the mRNA levels of *alpha-S1-casein* (*CSN1S1*), *alpha-S2-casein* (*CSN1S2*), *beta-casein* (*CSN2*), *kappa-casein* (*CSN3*), and *lactalbumin* (*LALBA*) were also detected. The result showed that the mRNA level of *CSN1S1*, *CSN1S2*, *CSN2*, *CSN3*, and *LALBA* significantly (*p <* 0.01) dropped (S4 Fig) in the *BLG* KO goat’s mammary glands.

To investigate the expression of BLG protein in kids’ milk, we performed SDS-PAGE with Coomassie blue staining. The Coomassie blue staining results of #B1-2 sample showed two unexpected bands indicating unknown proteins with a lower or a higher molecular weight, respectively (Fig 3C). Furthermore, western blot results showed that the BLG protein had been abolished in the milk of the *BLG* KO goat (Fig 3E).

In comparison with WT goats, fat, protein, lactose, and solid not fat in the milk of the *BLG* KO goat decreased by 7.68,7.97, and 7.71%, respectively (Table 2).

**Table 2:** Comparison of the components of milk from the bi-allelic *BLG* KO goat versus WT goats.

Animal	Fat (%)	Protein (%)	Lactose (%)	SNF (%)
*BLG* KO	4.65	4.45	3.35	8.51
WT	4.92±0.20	4.82±0.06	3.64±0.04	9.22±0.11

SNF, solid not fat.

### Chimerism analysis

**Fig 4:**
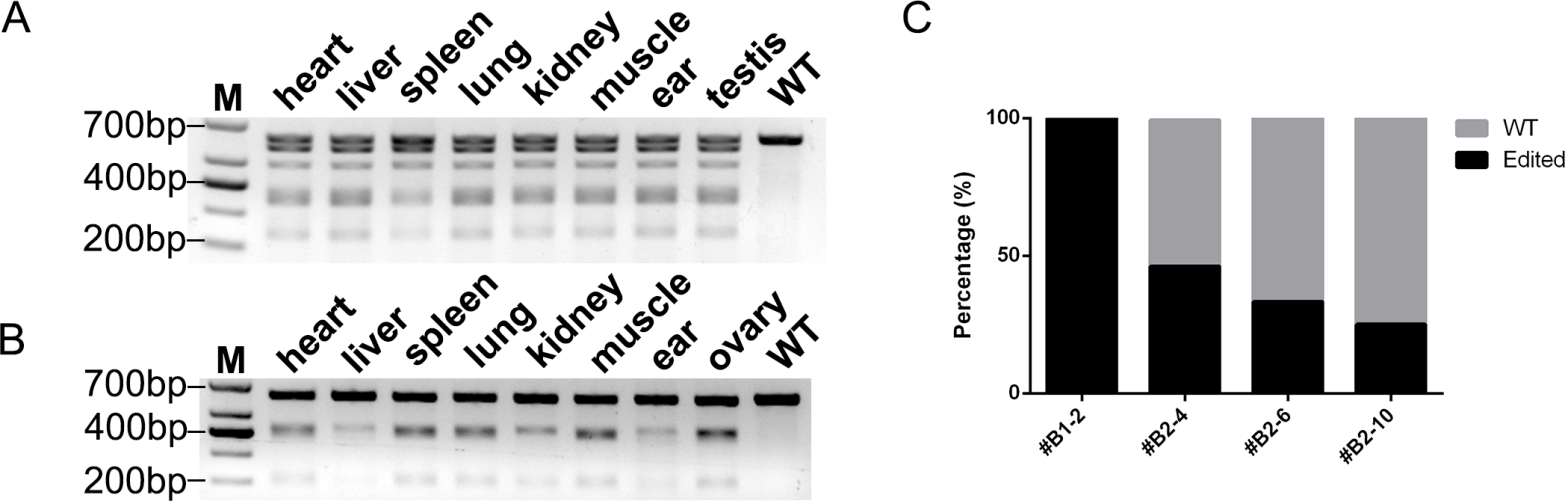
Chimerism analysis. (**A**) Detection of Cas9 editing in different tissues. Dead goat #B2-4 tissues were analyzed by the T7E1 assay. (**B**) Detection of Cas9 editing in different tissues. Dead goat #B2-10 tissues were analyzed by the T7E1 assay. (**C**) Analysis of the percentage of edited genome in *BLG* KO goats. The percentage of editing positive clones among TA clones of #B1-2 (n = 12), #B2-4 (n = 13), #B2-6 (n = 15), and #B2-10 (n = 12).

We counted the proportion of edited clones in targeted goat T-clone sequences and found that the edited clone proportion was less than 50%, except for #B1-2 (Fig 4C), suggesting that the modifications in the targeted kids are not mono-allelic but chimeric. To determine whether organs and tissues from CRISPR/Cas9 modified goats were edited simultaneously, we collected organs and tissues from dead targeted kids (#B2-4 and #B2-10) and extracted the genomic DNA. The target regions were amplified and subjected to the T7E1 assay. The results showed that all target sites in the different organs from two targeted kids were modified simultaneously, indicating that Cas9 editing occurred during early embryogenesis. (Fig 4A and 4B).

### OT analysis

OT effects are of concern to many researchers utilizing the CRISPR/Cas9 system [24]. We screened a total of nine potential OT sites, including each three for sg1, sg2, and sg3, to determine whether OT effects occurred (S2 Table). T7E1 cleavage assay results showed no detectable OT effects in our *BLG*-modified goats (S5 Fig).

## Discussion

BLG protein presents in milk from cows and goats but not in human milk, and therefore is a major allergen in cow or goat milk [25]. It can cause an intestinal allergic reaction, especially diarrhea in infants [26]. However, neither heat processing nor fermentation could eliminate BLG protein in milk [5, 27, 28]. Therefore, the generation of *BLG* KO livestock is necessary to evaluate the biology of BLG-free milk. In this study, we achieved locus-specific modification in goat fibroblasts using CRISPR/Cas9. We report a convenient way to generate *BLG* KO goats by co-injection of Cas9 mRNA and sgRNAs in goat one-cell-stage embryos.

We first tested the efficiency of CRISPR/Cas9 system in goat fibroblasts. Sequencing results showed a low editing activity for single sgRNA-guided targeting (8.00%-9.09%, Fig 1), which may be due to the low transfection efficiency (S1 Fig). It is reported that a high cleavage activity (9–70%) was achieved in goat fibroblasts by electroporation [13]. Wang achieved approximately 50–75% editing efficiency for single sgRNA cleavage and 61.11% efficiency for dual sgRNA-guided cleavage using Blasticidin to eliminate cells that were transfected unsuccessfully [16]. Therefore, CRISPR/Cas9 can be successfully applied in goat cell genome editing.

To determine whether we could generate *BLG* KO goats through co-injection of Cas9 mRNA and sgRNAs into one-cell-stage embryos, we determined the injection concentration of Cas9 and sgRNA according to Huang’s group [29] at the first round of injection because the low concentration of Cas9 mRNA and sgRNA they used may reduce the off-target activity. As a result, poor single sgRNA-targeting efficiencies (12.5 and 0%) were obtained, and the low concentrations of Cas9 mRNA and sgRNA (Table 1) could have been the primary reason. However, some studies have reported that dual sgRNA targeting might increase the efficiency of modification and cause large fragment deletions [30]. Also, low toxicity has been reported for high concentrations of Cas9 mRNA and sgRNA in embryos [18, 31]. Therefore, we co-injected dual sgRNAs with Cas9 mRNA to improve the targeting efficiency. As summarized in Table 1, the targeting efficiencies of the 50 ng/μL sg2+sg3 group (25.0%) and 100 ng/μL sg2+sg3 group (28.6%) are similar to that reported in other studies using the injection method (15–59%) [16, 18]. Furthermore, some studies reported that the editing efficiency could even be 80%-100% in mice and rabbits [32, 33]. Thus, co-injection of embryos with sgRNAs and Cas9 mRNA is a convenient and less laborious method for generating genome-modified livestock.

In our study, a 29-bp deletion in *BLG* exon 1, including part of the signal peptide coding sequence, caused a frameshift mutation. The level of *BLG* expression dropped sharply (*p <* 0.01) in the *BLG* KO goats (Fig 3B), in agreement with reports from other transgenic goat studies [14]. As the Coomassie blue staining results showed, we hypothesized that a shorter peptide with mutations would be translated and that the expression of WT BLG protein in milk from the *BLG* KO goat would be abolished, which could explain the disappearance of 18-kDa WT BLG band in the *BLG* KO goat milk sample (Fig 3C). Furthermore, the western blot result confirmed the lack of WT BLG protein in the *BLG* KO goat milk (Fig 3E).

The specificity of the CRISPR/Cas9 system is of significant concern to researchers. Our OT detection showed no OT activity at the predicted loci. Recent *in vitro* studies suggested that mismatches beyond the seed sequence (8- to 12-bp nucleotides adjacent to protospacer adjacent motifs) can be tolerated [24, 34]. Although a few OT activities of Cas9 have been reported [35, 36], many other studies in livestock have shown a high fidelity of Cas9 [13, 16, 17, 32, 37, 38]. In addition, the use of Cas9 nickase (Cas9D10A or Cas9H840A) [39] or truncated sgRNAs [40] to reduce the occurrence of OT effects have been reported. An “enhanced specificity” SpCas9 (eSpCas9) with low OT activity has been developed, suggesting that Cas9 could be utilized for high-precision genome modification in animals and gene therapy [41].

In Table 2, we found a drop of total protein in the *BLG* KO goat’s milk. BLG accounts for about 50% of the whey protein in ruminants and nearly 10% of the gross protein in milk [42]. So, abolishment of BLG protein could possibly reduce the total protein in milk (Table 2, Fig 3B and 3E). It is reported that knocking out *CSN2* in mice led to decrease of total protein and increase of whey protein, and BLG concentration was negatively related to casein ratio in goat milk, suggesting a compensation effect between casein and whey proteins [43, 44]. Therefore, this compensation effect could possibly explain why the percentage of protein dropped less than 10% and the enhanced bands of caseins and other whey proteins in the SDS-PAGE result (Fig 3C). However, it needs to be further investigated whether the drop of milk protein coding genes’ expression was related to the knock-out of *BLG*.

In conclusion, we developed a convenient method for generating *BLG* KO goats through micro-injection of Cas9 mRNA and sgRNAs. Deletion of the BLG coding sequence induces decreases in both BLG mRNA expression and BLG protein concentration in mammary glands and milk. In addition, our study provides a basis for generating *BLG* KO goats and obtaining humanized milk.

## Supporting information

S1 Fig Cell transfection efficiencies of lipofection and electroporation. (PDF)

S2 Fig Mutations at the BLG CDS locus of targeted kids and their corresponding protein mutations. (PDF)

S3 Fig Comparison of heterozygous gDNA regions of goats with unexpected bands in T7EN1 assay. (PDF)

S4 Fig Milk protein coding gene expression in goat mammary gland. Expression levels of *CSN1S1*, *CSN1S2*, *CSN2*, *CSN3*, and *LALBA*.
(PDF)

S5 Fig Off-target assay of targeted goats at 9 OTs. (PDF)

S1 Table Details of gRNAs for goat BLG locus. (PDF)

S2 Table Potential off-target sites of sg1, sg2, and sg3. (PDF)

S3 Table Oligonucleotides used in this study. (PDF)
