# Supplementary material for: Generation of beta-lactoglobulin knock-out goats using CRISPR/Cas9
Source: PLoS One. 2017 Oct 10;12(10):e0186056. doi: 10.1371/journal.pone.0186056 (PMC5634636; doi:10.1371/journal.pone.0186056)
Supplement: S1 Fig — (PDF) [file pone.0186056.s001.pdf]

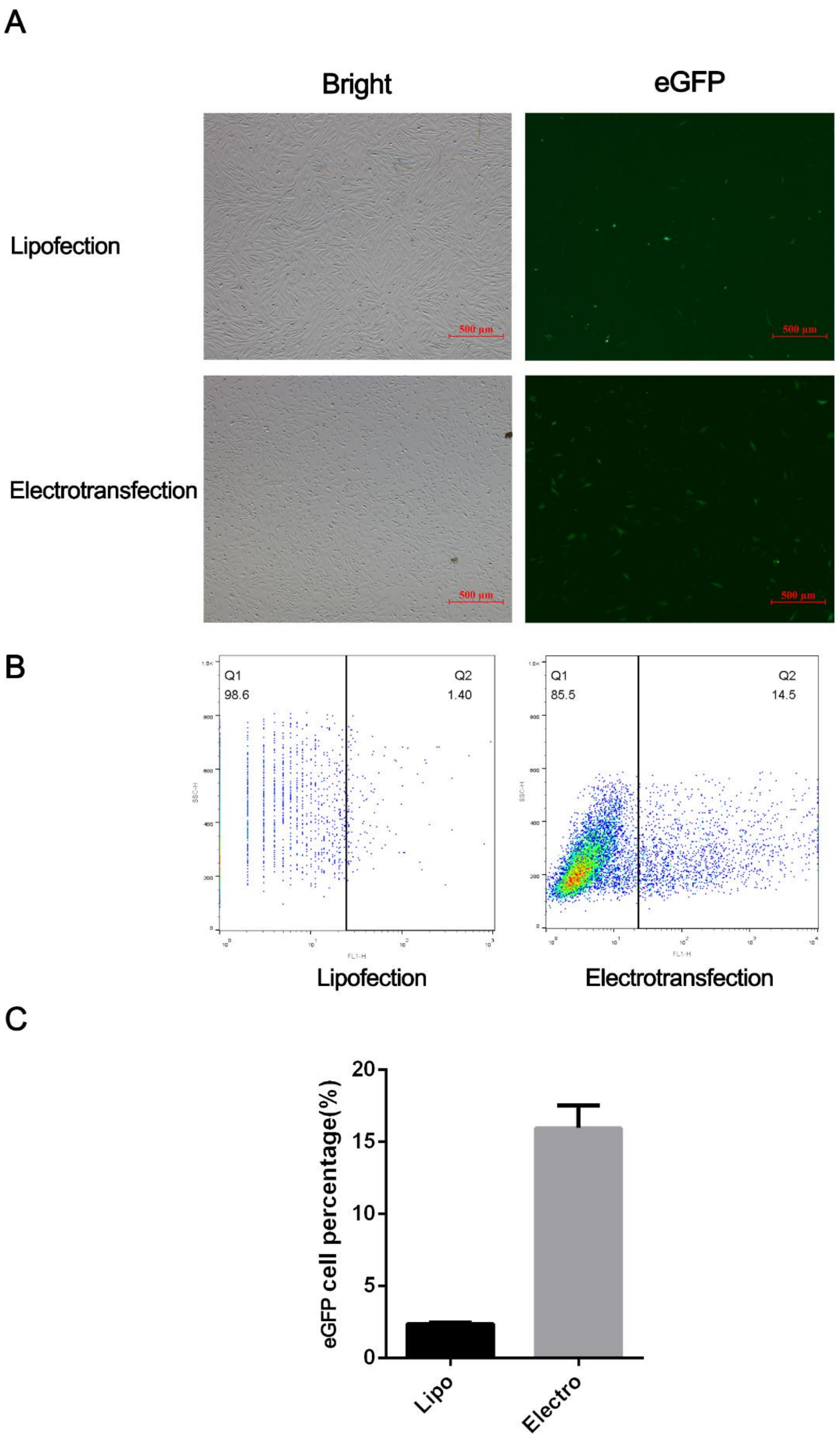

S1 Fig. Cell transfection efficiencies of lipofection and electroporation. (**A**) Photos of goat ear fibroblasts transfected with the eGFP plasmid 72 h after transfection. (**B**) Goat ear fibroblasts were transfected with the eGFP plasmid using lipofection or electroporation and analyzed by flow cytometry. (**C**) Transfection efficiencies of two different transfection methods.
