# Supplementary material for: Generation of beta-lactoglobulin knock-out goats using CRISPR/Cas9
Source: PLoS One. 2017 Oct 10;12(10):e0186056. doi: 10.1371/journal.pone.0186056 (PMC5634636; doi:10.1371/journal.pone.0186056)
Supplement: S2 Fig — (PDF) [file pone.0186056.s002.pdf]

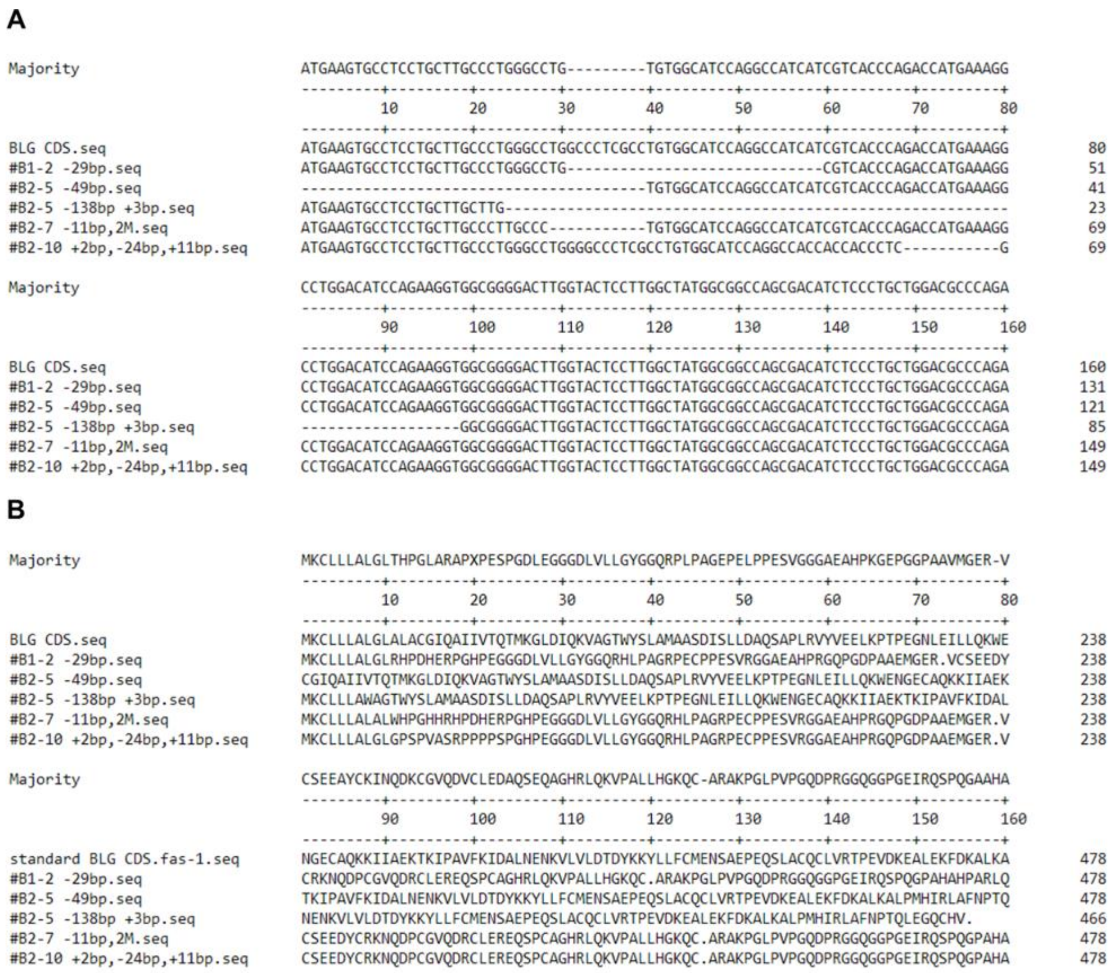

C

Indel type	Protein Mutation
#B1-2 -29bp.seq	Open reading frame disruption
#B2-5 -49bp.seq	Open reading frame disruption
#B2-5 -138bp +3bp.seq	Open reading frame disruption
#B2-7 -11bp,2M.seq	Open reading frame disruption
#B2-10 +2bp,-24bp,+11bp.seq	Open reading frame disruption

S2 Fig. Mutations at the BLG CDS locus of targeted kids and their corresponding protein mutations. (**A**) Multi-alignment of five mutations. (**B)** Correspondence mutations of protein sequences according to **A**. (**C)** Summary of protein mutation types.
