# Supplementary material for: Generation of beta-lactoglobulin knock-out goats using CRISPR/Cas9
Source: PLoS One. 2017 Oct 10;12(10):e0186056. doi: 10.1371/journal.pone.0186056 (PMC5634636; doi:10.1371/journal.pone.0186056)
Supplement: S3 Fig — (PDF) [file pone.0186056.s003.pdf]

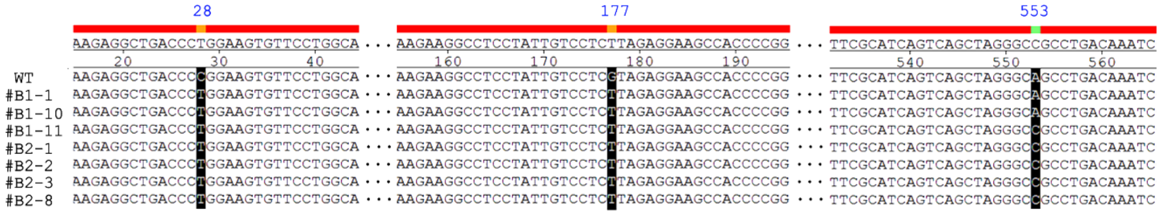

S3 Fig. Comparison of heterozygous gDNA regions of goats with unexpected bands in T7EN1 assay. Different genotypes were found in the sequencing results of goats with unexpected bands in the T7EN1 assay. Goats (#B1-1, #B1-10 and #B1-11) with two SNPs were expected to have a 28-bp, a 149-bp and a 445-bp band in the T7EN1 assay results. And goats (#B2-1, #B2-2, #B2-3 and #B2-8) with three SNPs were expected to have a 28-bp, a 149-bp, a 376-bp and a 445-bp band in the T7EN1 assay results.
