# Supplementary material for: Generation of beta-lactoglobulin knock-out goats using CRISPR/Cas9
Source: PLoS One. 2017 Oct 10;12(10):e0186056. doi: 10.1371/journal.pone.0186056 (PMC5634636; doi:10.1371/journal.pone.0186056)
Supplement: S4 Fig — Expression levels of CSN1S1, CSN1S2, CSN2, CSN3, and LALBA. (PDF) [file pone.0186056.s004.pdf]

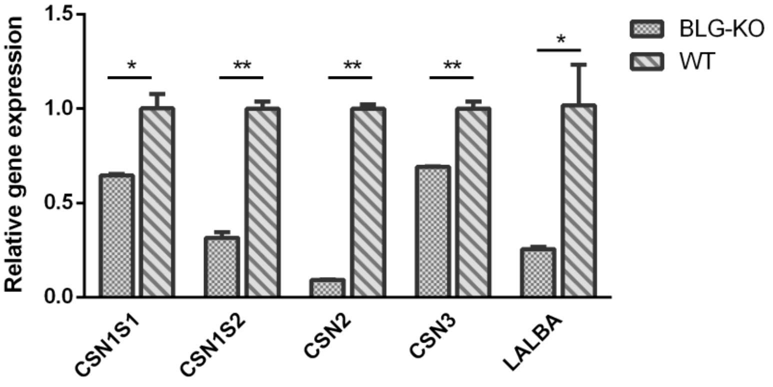

S4 Fig. Milk protein coding gene expression in goat mammary gland. Relative expression of major milk genes except *BLG*. Values with one asterisk ($p < 0.05$) or two asterisks ($p < 0.01$) are significantly different. Three assay replicates were performed for each tissue sample. BLG-KO, #B1-2. WT, wildtype goats. *CSN1S1*, *CSN1S2*, *CSN2*, *CSN3*, and *LALBA* are symbols of *alpha-S1-casein*, *alpha-S2-casein*, *beta-casein*, *kappa-casein*, and *lactalbumin* respectively.
