# Supplementary material for: Generation of beta-lactoglobulin knock-out goats using CRISPR/Cas9
Source: PLoS One. 2017 Oct 10;12(10):e0186056. doi: 10.1371/journal.pone.0186056 (PMC5634636; doi:10.1371/journal.pone.0186056)
Supplement: S5 Fig — (PDF) [file pone.0186056.s005.pdf]

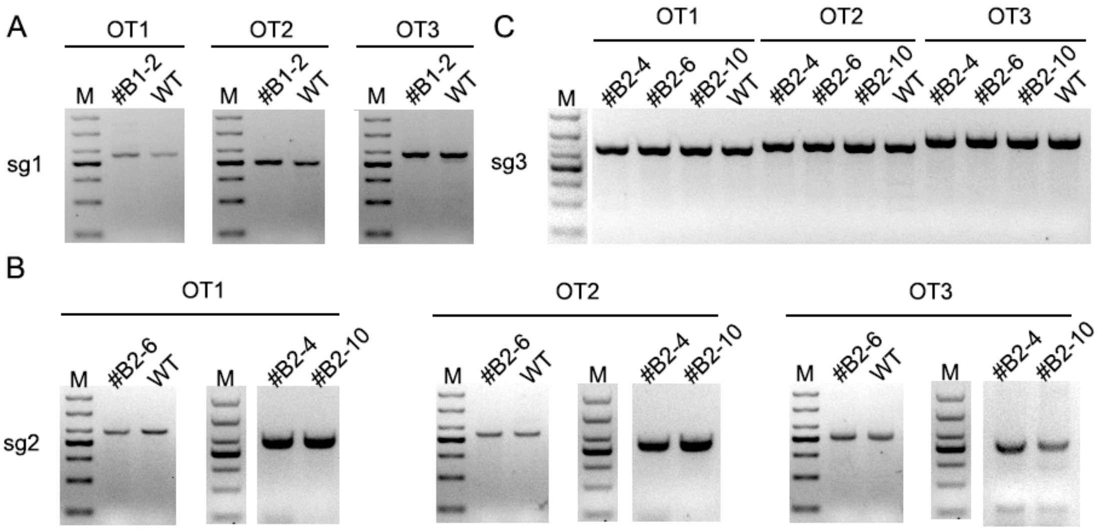

S5 Fig. Off-target assay of targeted goats at 9 OTs. (**A**) T7E1 cleavage assay of 3 potential OTs of sg1 (**B**) T7E1 cleavage assay of 3 potential OTs of sg2 (**C**) T7E1 cleavage assay of 3 potential OTs of sg3
