# Supplementary material for: Generation of beta-lactoglobulin knock-out goats using CRISPR/Cas9
Source: PLoS One. 2017 Oct 10;12(10):e0186056. doi: 10.1371/journal.pone.0186056 (PMC5634636; doi:10.1371/journal.pone.0186056)
Supplement: S1 Table — (PDF) [file pone.0186056.s006.pdf]

S1 Table. Details of gRNAs for goat.

sgRNA	Gene target	Target site sequence (5`→3`)
sg1		GGCCCTCGCCTGTGGCATCCAGG
sg2	BLG Exon1	GGATGCCACAGGCGAGGGCCAGG
sg3		ATCGTCACCCAGACCATGAAAGG
