# Supplementary material for: Generation of beta-lactoglobulin knock-out goats using CRISPR/Cas9
Source: PLoS One. 2017 Oct 10;12(10):e0186056. doi: 10.1371/journal.pone.0186056 (PMC5634636; doi:10.1371/journal.pone.0186056)
Supplement: S2 Table — (PDF) [file pone.0186056.s007.pdf]

S2 Table. Potential off-target sites of sg1, sg2, and sg3.

sgRNA	OTs	sequence (5`→3`)	Mismatches	Chromosome
sg1	OT1	GGCCCTCGgCaGTGGCATCCTGG	2MMs[9,11]	chr11
	OT2	GGCCCTCcCCTcTGGCATCCCAG	2MMs[8,12]	chrX
	OT3	tGCCCTCctCTGTGGCATCCAGG	3MMs[1,8,9]	chr1
sg2	OT1	GGgTGCCACAGGtGAGGGCCAAG	2MMs[3,13]	chr11
	OT2	GtATGCCACAGGaGAGGGCCAGG	2MMs[2,13]	chr13
	OT3	GGATGCCAgAGGgGAGGGCCAGG	2MMs[9,13]	chrX
sg3	OT1	ATaGaCACCaAGACCATGAAAGG	3MMs[3,5,10]	chr6
	OT2	gTtGTCACCCAGtCCATGAACAG	3MMs[2,3,9]	chr10
	OT3	AgCGTCAggCAGtCCATGAAGGG	4MMs[2,8,9,13]	chr11

The lowercase letter suggests a mismatch to the sgRNA target site.
