# Supplementary material for: Generation of beta-lactoglobulin knock-out goats using CRISPR/Cas9
Source: PLoS One. 2017 Oct 10;12(10):e0186056. doi: 10.1371/journal.pone.0186056 (PMC5634636; doi:10.1371/journal.pone.0186056)
Supplement: S3 Table — (PDF) [file pone.0186056.s008.pdf]

S3 Table. Oligonucleotides used in this study.

Oligonucleotides used for pX330-gRNA plasmid construction.

sgRNA	Direction	Oligoes (5`→3`)
sg1	F	caccGGCCCTCGCCTGTGGCATCC
	R	aaacGGATGCCACAGGCGAGGGCC
sg2	F	caccGGATGCCACAGGCGAGGGCC
	R	aaacGGCCCTCGCCTGTGGCATCC
sg3	F	caccGATCGTCACCCAGACCATGAA
	R	aaacTTCATGGTCTGGGTGACGATC

Oligonucleotides used for amplification of sgRNA-targeting region.

Primers	Direction	Oligoes (5`→3`)	Products length (bp)
T7-BLG	F	ACCTGCCCTTGTCTAAGA	622
	R	CTTCCAGCCTCCAGAATG	

Oligonucleotides used for PCR amplification of templates for in vitro transcription.

Primers	Sequences (5`→3`)
IVT_Cas9_F	taatacgactcactatagggAGAATGGACTATAAGGACCACGAC
IVT_Cas9_R	GCGAGCTCTAGGAATTCTTAC
IVT_sg1	ttaatacgactcactatagGGCCCTCGCCTGTGGCATCC
IVT_sg2	ttaatacgactcactatagGGATGCCACAGGCGAGGGCC
IVT_sg3	ttaatacgactcactatagGATCGTCACCCAGACCATGAA
IVT_gRNA_R	AAAAGCACCGACTCGGTGCC

Oligonucleotides used for qPCR amplification

Primers	Gene ID	Direction	Sequences (5`→3`)
CSN1S1	NM_001285695.1	F	CCTAATCCCATTGGCTCTGA
		R	TGACTCTTCACCACAGTGGC
CSN1S2	NM_001285585.1	F	CAGGTTAAGAGAAATGCTGGCC
		R	TGGTGGAGAGCTGCTCTCTGTT

CSN2	NM_001081852.1	F	CCTCTTACTCAAACCCCTGTGG
		R	GGACTCCCATTATTTCAGGCTG
CSN3	NM_001285587.1	F	CCTGCCATCAATACCATTGCT
		R	TGCTTCGGTGGTAGGTGTACTG
LALBA	NM_001285635.1	F	CACCTGCTGTCTTTGCTGCTT
		R	CACCTGCTGTCTTTGCTGCTT
BLG	NM_001285539.1	F	GATCCCTGCGGTGTTCAAGAT
		R	CAGCACTGTTTTCCATGCAGAA
GAPDH	XM_005680968.3	F	AACGTGTCCGTTGTGGATCT
		R	GAGTGTCGCTGTTGAAGTCG

CSN1S1, *CSN1S2*, *CSN2*, *CSN3*, *LALBA* and *BLG* are symbols of *alpha-S1-casein*, *alpha-S2-casein*, *beta-casein*, *kappa-casein*, *lactalbumin* and *beta-lactoglobulin* respectively.

Oligonucleotides used for off-target activity detection

sgRNA	OTs	Direction	Sequences (5`→3`)	Product Length (bp)
sg1	OT1	F	TGATAGTCAGGTGGGAGGGTT	401
		R	AACCCCAGGTGGGTGAGTG	
	OT2	F	TTTTCATGGCAGAGCCAGCAT	461
		R	AAAGGCTCGTAGCTTCTGGAG	
	OT3	F	CGACTGTCCCACAAGGTTTGA	485
		R	ATTCTGGCCAAGTGGTACTGG	
sg2	OT1	F	AAGTGTCCTGGTGCTGCTC	409
		R	CCTCAGCCTCAGGCCC	
	OT2	F	AGCAAAGCACTTTCAGCCCC	488
		R	CGCCCATTTCCTGTACCTGG	
	OT3	F	CCAGCATTTGAACCTCTCCCT	447
		R	AAAGGCTCGTAGCTTCTGGAG	
sg3	OT1	F	TATGGAGGCTGACCAAACCA	575
		R	ACTCAACAGTTAGCCTTTCGG	
	OT2	F	ACCCTCTTCCGCCATAGTCA	699
		R	AGAGGCAACACCATCCAAGT	
	OT3	F	TCCCTATTTGATAAATCCCCCAGA	621
		R	TGTGCCAGCCAGGACTATTTT	
